# Hospital Meetings, &c.

**Published:** 1897-08-14

**Authors:** 


### HOSPITAL MEETINGS, &C.

### ROCHDALE INFIRMARY—OPENING THE HOLDEN WING.

The extension of the infirmary known as the Holden wing was formally opened on July 24th amidst great rejoicings. The streets were lavishly decorated, and a long procession escorted Lord Stanley of Knowsley to the infirmary, where several ladies and gentlemen were introduced to him and a golden key presented. The funds for the endowment were aided by the splendid donation of £18,000 by the trustees of the late Mr. James Holden, of Marland, and the sum of £6,000 collected locally in honour of the Queen's long reign. The Holden wing is connected with the old infirmary from the east side of an old ward by a door opening into the new hall, from which the bathrooms, kitchens, and men's ward are accessible. Accommodation is offered in this ward for 12 male patients, and it is fitted with the latest improvements. The walls are tiled, and the corners are rounded, the flooring is of pitch pine, especially laid, and great attention has been paid to adequate heating and ventilation. The children's ward is immediately over this, ward and is similarly fitted. There is also an isolation ward of four beds at some little distance from the main block. The accommodation for out-patients is arranged in the basement, and is quite separate from that prepared for in-patients. The endowed beds were unveiled on this occasion, and many congratulatory speeches were made at the luncheon in the Town Hall after the ceremony.

